# Let’s Print an Ecology in 3D (and 4D)

**DOI:** 10.3390/ma17102194

**Published:** 2024-05-07

**Authors:** Magdalena Szechyńska-Hebda, Marek Hebda, Neslihan Doğan-Sağlamtimur, Wei-Ting Lin

**Affiliations:** 1 W. Szafer Institute of Botany Polish Academy of Sciences, Lubicz 46, 31-512 Kraków, Poland; 2 Faculty of Materials Engineering and Physics, Cracow University of Technology, Warszawska 24, 31-155 Kraków, Poland; marek.hebda@pk.edu.pl; 3 Department of Environmental Engineering, Nigde Omer Halisdemir University, Niğde 51240, Türkiye; neslihandogansaglamtimur@gmail.com; 4 Department of Civil Engineering, National Ilan University, No. 1, Sec. 1, Shennong Rd., I-Lan 260, Taiwan; wtlin@niu.edu.tw

**Keywords:** additive manufacturing, ecology, materials, sustainability

## Abstract

The concept of ecology, historically rooted in the economy of nature, currently needs to evolve to encompass the intricate web of interactions among humans and various organisms in the environment, which are influenced by anthropogenic forces. In this review, the definition of ecology has been adapted to address the dynamic interplay of energy, resources, and information shaping both natural and artificial ecosystems. Previously, 3D (and 4D) printing technologies have been presented as potential tools within this ecological framework, promising a new economy for nature. However, despite the considerable scientific discourse surrounding both ecology and 3D printing, there remains a significant gap in research exploring the interplay between these directions. Therefore, a holistic review of incorporating ecological principles into 3D printing practices is presented, emphasizing environmental sustainability, resource efficiency, and innovation. Furthermore, the ‘unecological’ aspects of 3D printing, disadvantages related to legal aspects, intellectual property, and legislation, as well as societal impacts, are underlined. These presented ideas collectively suggest a roadmap for future research and practice. This review calls for a more comprehensive understanding of the multifaceted impacts of 3D printing and the development of responsible practices aligned with ecological goals.

## 1. Introduction

Novel technologies such as 3D printing represent significant milestones for diverse industries and scientific disciplines. These technologies hold immense promise in reshaping manufacturing processes, enabling the fabrication of complex structures with unprecedented precision and efficiency. 3D printing revolutionized conventional manufacturing paradigms by offering unparalleled design flexibility, reduced lead times, and minimized material wastage. The applications of additive manufacturing span a wide spectrum of industries, from aerospace components to biomedical implants, each poised to benefit from its transformative capabilities [1,2,3,4,5,6].

However, given the relentless advancement of these technologies, it becomes increasingly apparent that their adoption presents formidable challenges within ecology and sustainability [7]. A complex interplay between innovation and ecological stewardship, including resource consumption, the energy-intensive nature of additive manufacturing processes, and end-of-life management, underscores the urgent need for a holistic assessment of ecological footprints.

This review considers the significant impact of humans on the environment and the increasing control exerted over natural systems through anthropogenic interventions related to new technologies, with a focus on technologies such as 3D and 4D printing. In this context, the ambiguous title ‘LET’S PRINT AN ECOLOGY IN 3D’ highlights the responsibility humans have for their long-term actions, extending beyond maintaining balance in the natural environment. Its ambiguity arises from the potential for a more literal interpretation, such as a physical creation or reproduction of the elements of or a whole ecological system using 3D printing technology. It can potentially be interpreted in a more metaphorical way, as an invitation to use 3D printing technology to create environmentally friendly or sustainable products and solutions that contribute positively to ecology or environmental conservation efforts. Moreover, the phrase can be seen as provocative due to its potential for multiple connotations. Firstly, it possibly presents an unconventional idea of creating or replicating an ecology through 3D printing. It challenges traditional notions of ecology as something natural and complex, implying that it could be artificially constructed, and thus it raises questions about the distinction between what is natural and what is artificial. It also challenges the notion of authenticity in natural systems and prompts readers to reflect on the extent to which humans are manipulating and reshaping the environment. Secondly, it brings together the two seemingly disparate realms of technology (represented by 3D printing) and nature (represented by ecology). It suggests a shift towards anthropogenic solutions to ecological problems, instead of relying on natural processes that have evolved over millions of years. It also implies a preference for interventions designed by humans, and thus the ethical and environmental implications. Thirdly, it could be interpreted as a call to action, challenging individuals or society as a whole to consider new ways of engaging with and understanding ecology, or it might encourage reflection on the extent to which humans are shaping and altering the natural world through technological interventions.

By connecting the potential applications of 3D and 4D printing in the context of human impacts on natural systems, this review bridges the gap between material technology and ecology. It highlights the multifaceted interpretations of the phrase ‘LET’S PRINT AN ECOLOGY IN 3D’, delving into its literal and metaphorical implications for sustainable innovation. Through a synthesis of a wide range of publications from different disciplines and topics, empirical research, theoretical frameworks, and practical insights, we seek to elucidate the intricate nexus between technological innovation and environmental sustainability. Analyzing the advantages and disadvantages of 3D printing, along with the ecological challenges posed by emerging technologies, we propose strategies for mitigating negative environmental impacts.

## 2. Ecology Definition: The Economy of Nature

The term ‘ecology’ was coined in 1866 by the German zoologist Ernst Haeckel to describe the ‘economy of living forms’, ‘the study of the interrelationships of organisms with their environment and each other’, and ‘the biology of ecosystems’. He derived the word ‘ecology’ from the Greek words ‘oikos’, meaning ‘household’, and ‘logos’, meaning ‘study’ or ‘science’. Thus, ‘oekologie’ originally referred to the relationships between organisms and their physical (inorganic) environment, including factors related to climate, soil, water, and other abiotic factors [8,9]. Over time, the definition and scope of ecology have expanded and evolved. In 1942, Lindeman published ‘The Trophic-Dynamic Aspect of Ecology’ [10], in which he introduced the trophic-dynamic concept of ecosystem ecology and outlined the flow of energy through ecosystems, emphasizing the transfer of energy from producers (such as plants) to consumers (such as herbivores, carnivores, and decomposers) and the subsequent cycling of resources. The statement of the Ecological Society of America (ESA) widened the definition of ‘ecology’ by emphasizing the connections between plants, animals, and their environment for the sustainable use of Earth’s resources. Encyclopædia Britannica [11] also acknowledges that the most pressing problems in human affairs have ecological dimensions. This recognition highlights the interconnectedness between human societies and the natural world; the significant influence of ecological factors on human well-being and sustainability; and ecological problems, such as climate change, expanding populations, biodiversity loss, pollution, habitat destruction, and resource depletion [8,12,13,14].

Based on the challenges outlined, the concept of ‘ecology’ and thus its current definition should encompass the intricate network of interactions and interdependencies between humans and other living organisms (plants, animals, microorganisms, etc.) in their shared environment. One approach to revising the definition of ecology involves considering the dynamic interplay of natural and anthropogenic forces, which collectively shape and regulate the flow of energy, resources, and information within complex systems. This comprehensive perspective needs deep research to capture the multifaceted interactions within ecosystems and emphasizes the interconnectedness of biotic and abiotic components. The starting point of this concept involves research acknowledging the delicate balance and resilience of natural systems, which have evolved to sustain life on Earth over millions of years. Energy flows through ecosystems in a one-way direction, primarily driven by the sun’s energy and photosynthesis, and is transferred among organisms within food chains. Sunlight, temperature, and climate patterns influence the availability and distribution of energy within ecosystems, while anthropogenic habitat destruction, pollution, and climate change can disrupt energy flows. Resource flows such as nutrient cycling, hydrological (water) cycles, and geological (mineral) processes regulate the availability and distribution of resources within ecosystems [8,9]. The next step involves researching the impact of human activities within the context of the Economy of Nature, recognizing the profound influence of human actions on ecological processes and dynamics. Human activities can disrupt such cycles through deforestation, pollution, over-extraction of water, nutrient runoff from agriculture, etc. This highlights the consequences of resource extraction, pollution, habitat destruction, climate change, and the introduction of invasive species, which disrupt the natural functioning of ecosystems and threaten biodiversity and ecosystem health. Information flows through ecosystems, including communication among organisms, chemical signaling, and ecological interactions that force biological behavior, genetic variation (acclimation, adaptation), and ecological succession (dynamics). Anthropogenic forces can impact the flow of information through disruptions to communication networks, the introduction of novel stressors, and the alteration of ecological interactions. Further, the dynamic interplay of natural and anthropogenic factors that drive the flow of energy, resources, and information within ecosystems necessitates a comprehensive understanding of effective ecosystem management, conservation efforts, sustainable development, ecosystem health and resilience, and the well-being of human societies [8,13,14]. However, by accounting for these forces, this approach offers a more nuanced understanding of the processes that govern ecological systems and their responses to environmental changes. Finally, by embracing changes in these interactions, we can better anticipate future ecological challenges within ecosystems.

## 3. 3D (and 4D) Printing: The Economy for Nature

### 3.1. History: Bridging Innovation with Environmental Sustainability

The evolution of the additive manufacturing industry has paved the way for advancements in 3D and 4D printing technologies since 1980 [15,16,17], when Hideo Kodama patented rapid prototyping using UV rays and resin. Four years later, Chuck Hull revolutionized the field with the invention of stereolithography (SLA), a process that solidifies photopolymer layers. In 1988, Carl Deckard developed the selective laser sintering (SLS) process for powder materials and SLS printers, whereas Scott Crump and Lisa Crump established fused deposition modeling (FDM), a process that extrudes thermoplastic material to build up layers. Concurrently, in the realm of metal 3D printing, Hans J. Langer introduced the concept of metal laser sintering (MLS), and sold the world’s first stereo system for metal 3D printing. However, the most significant boost to the 3D printing industry came in the early 2000s with the emergence of open-source projects such as the RepRap Movement and Fab@Home. These initiatives aimed to democratize 3D printing by sharing designs and knowledge with individuals worldwide, fueling a wave of innovation and experimentation in the field.

The initial focus on pioneering methods has quickly expanded to encompass a variety of practical applications in different disciplines. This progression, coupled with the open-source initiatives, encouraged innovation and experimentation. However, despite the substantial volume of scientific documents published in the fields of ecology and 3D printing (951,020 and 127,494 documents found in the Scopus database, respectively), there remains a notable gap in research concerning the utilization of ecology in 3D printing methods and 3D printing for ecology. Table 1 presents the distribution of documents available for the specified keywords: ‘3D AND print* AND ecolog*’ (total of 461 documents found) or ‘3D AND print* AND ecology’ (total of 162 documents found) in various subject areas of the Scopus database (Table 1). The subjects, categorized into distinct academic fields, showed that Environmental Science, Social Sciences, Engineering, and Computer Science are fields related to ‘3D printing and ecology’, collectively covering almost 60% of the published papers. By integrating ecological considerations into 3D printing practices, these disciplines contribute to the advancement of sustainable development and environmental conservation.

### 3.2. Modern Trends and Valuable Investments: Where 3D Printing Meets Ecology

**Figure 1:**
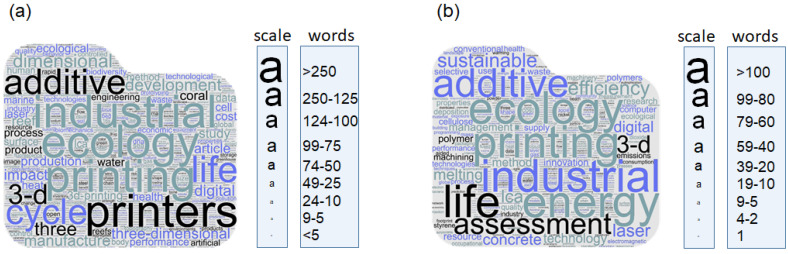
Detailed topics of publications in the Scopus database according to the search for: ‘3D AND print* AND ecolog*’ ((**a**) total of 461 publications found) or ‘3D AND print* AND ecology’ ((**b**) total of 162 publications found). Author keywords and indexed keywords for the publications with the highest citation record (top 10%; citation index from 43 to 1025) were exported as plain text, cleaned from descriptors, and are presented as word cloud graphs. The size of the letters indicates the frequency of a particular keyword within the exported publications. Search date: 22 March 2024. https://www.wordclouds.com/ was used to create the graphics.

The connection between 3D printing and ecology showcases the potential for innovative strategies to tackle ecological challenges. Although there are promising applications of 3D and 4D printing technologies within an ecological framework, the adoption of a comprehensive approach prioritizing environmental sustainability, resource efficiency, adaptation, and innovation remains uncommon. Taking into account the number of citations for individual publications, the most popular topics concerning 3D printing in an ecological context can be clustered into three main groups (Figure 1; Table 2), i.e., (1) related to modelling approaches [16,18,19,20] and developing new methods [21,22,23,24,25]; (2) related to environment such as smart farming and agriculture, including agriculture 4.0 [26], and the environmental impacts of additive manufacturing, including life-cycle assessment [27,28,29,30,31,32,33,34,35,36,37,38,39,40,41,42,43]; and (3) new printed materials for different industries and their physical, chemical, and mechanical properties [17,44,45,46,47,48,49], including those materials with natural (organic) origins [50,51,52,53] and waste origins [54].

Overall, each group of topics concerning 3D printing in an ecological context has the potential to contribute to a more sustainable manufacturing industry by reducing material and energy use and carbon emissions [6,55,56,57]; conserving resources [7,58,59], repairing and upcycling [56,60,61,62] and reducing waste generation [63,64,65,66]; facilitating the selection of eco-friendly materials [67,68,69,70,71]; and enabling decentralized and customized production processes [72,73,74], thus contributing to holistic assessments of environmental impacts.

In the group of publications related to modelling, there is a significant emphasis on understanding printing conditions and the properties of various printed materials. This knowledge aids in optimizing the printing process, resulting in more efficient resource use and waste reduction, thereby supporting ecological sustainability. These models allow researchers and practitioners to precisely adjust parameters for specific applications, enhancing both the quality and sustainability of 3D printing outcomes. The pioneering research on this subject was provided by Charles W. Hull, co-founder of 3D Systems, who outlined the process of using computational geometry modelling for layer-by-layer construction of 3D objects (‘Apparatus for production of three-dimensional objects by stereolithography’, US Patent 4,575,330, filed in 1984). The groundwork for applying topology optimization in various engineering applications, including additive manufacturing, was laid by Ole Sigmund (professor at the Technical University of Denmark), first mentioned in the seminal work ‘Topological design of structures and composite materials with multi-objectives’ [75]. Many other scientists and researchers, such as Hod Lipson, Neil Gershenfeld, Terry Wohlers, Elaine Cohen, and Jean-Claude André, have also made significant contributions, advancing various aspects of 3D printing through their work on specific mathematical modelling techniques. Some current applications involve characterizing the shear thinning behavior of inks by quantifying the degree of shear thinning and using mathematical models to predict the window of printer operating parameters within which the materials could be printed. Furthermore, the model predicted residence times for living cells at optimized printing conditions [76]. Adaptive Multi-Layer Customization (AMC), adaptive Generative Adversarial Networks (GAN), and mathematical models were used to optimize energy efficiency for eco-friendly 3D printing. Xu et al. [77] analyzed energy and material consumption, considering thermal, mechanical, and auxiliary subsystems. Customization parameters such as layer thickness and infill patterns improved energy efficiency by up to 11.51% and carbon emission reduction by up to 49.91%. Predicting the acoustical properties of materials using a matrix of impulse responses and mode interpolation [78] or the quality of electronics structures manufactured by a 3D inkjet printing process [79,80] was performed with different mathematical models to limit experimental trials save energy and material use during the process. More recently, artificial intelligence, machine learning, and deep learning have become integral components of 3D printing used in various aspects of additive manufacturing, including design optimization, predicting 3D printing parameters and process control, material development, part orientation, support generation, defect detection, quality control, etc. The application and importance of AI methods in 3D printing show promising advancements in eco-friendly applications, spanning from manufacturing to healthcare [81,82,83,84], e.g., in the machining industry [85], diagnosis systems to address anomalies and reducing printing errors [86,87], building reconstruction [83], predicting 3D-printed biomedical microneedle features [88,89], printable biomaterials [4,90,91], and automated and personalized production processes for pharmaceutics [92].

In the group of publications related to 3D printing methods, advancements in various techniques such as Stereolithography (SLA), Digital Light Processing (DLP), Liquid Crystal Display (LCD), Continuous Liquid Interface Production (CLIP), MultiJet Printing (MJP), two-photon 3D printing, and holographic 3D printing have emerged. These methods boast high precision, smooth surfaces, and fast printing speeds, making them suitable for efficient applications in biomedical fields, microfluidics, and soft robotics [93,94]. Moreover, the utilization and design of innovative materials like graphene composites and smart materials containing nanoparticles and fibers have necessitated the development of new methods and the optimization of existing ones (e.g., Fused Deposition Modeling (FDM), Direct Ink Writing (DIW), Stereolithography (SLA), and Selective Laser Sintering (SLS)). These advancements aim to create intricate structures and enhance material properties across various domains such as biomedical, mechanical, electrical, thermal, and optical industries [1,5,15,95].

In the group of publications addressed to environmental challenges within 3D printing technology, the studies aim to reduce material waste and environmental pollution. These efforts include developing biodegradable materials for 3D printing, methods for recycling materials like plastics, metals, and ceramics, and investigations into their mechanical properties after multiple recycling cycles [96]. Other studies have specialized in optimizing printing processes to minimize energy consumption and emissions, and reduce the environmental footprint of printed products [97,98,99], thus promoting sustainability [77,100,101]. Applying 3D printing technologies opens up new possibilities for smart farming and precision agriculture, such as increased nutrition value of final products, reducing food wastage [102]. Robots based on 3D printer ideas can seed plants, kill weeds, sense soil-moisture content, and irrigate plants individually over raised bed areas [103]; the design of eco-friendly advanced soft electronic devices (biocompatible and biodegradable) allows for environmental monitoring (sensors for measuring soil moisture level and temperature), grippers used in harvesting the crops, artificial lighting control, and energy harvesting and storage [26,104,105]. Since every material, product, and production process has an ecological impact, 3D printing studies often involve the Life Cycle Assessment of prototypical printing to establish a point of comparison to the environmental impact [106,107,108].

The group of publications on new 3D-printed materials emphasizes the significant impact that material types and their applications can have on ecology. This area of study is among the most researched, with a focus on investigating material properties such as biodegradability, recyclability, and mechanical performance. A wide variety of materials can be used for 3D printing, each with its own unique properties and applications, e.g., plastics, metals, composites, biomaterials, synthetic polymers, building material, ceramics, and even food (Table 2). Many new 3D printing materials are being developed from renewable and sustainable sources such as bioplastics [109], biomaterials (e.g., biomass–fungi biocomposite, modified cellulose, seaweed biopolymers [110,111,112], and bio-based resins [113]). By reducing reliance on fossil fuels and non-renewable resources, as well as due to their biodegradability or recyclability, these materials contribute to ecological sustainability by minimizing environmental impact and promoting circular economy principles. Tailoring the properties of 3D printing materials to specific applications (functional properties) can lead to more efficient and sustainable solutions, e.g., lightweight and high-strength materials can reduce material usage and energy consumption in transportation and aerospace applications, conductive and sensor-integrated materials enable smart and energy-efficient systems for environmental monitoring and control, and bioinspired lightweight composites can mimic the structure and properties of natural water filtration membranes [114,115,116,117]. The primary focus lies in the development of biomaterials suitable for 3D printing, e.g., biocompatible poly(ethylene glycol)diacrylate/nano-hydroxyapatite composites for continuous liquid interface production [118]; colloidal biomaterials using photo-reactive gelatin nanoparticles, showcasing the control over architecture and properties of biomaterial constructs [119]; capillary alginate gel for 3D-printing biomaterial inks to facilitate the integration, infiltration, and vascularization of 3D-printed structures [120]; poly(octamethylene maleate (anhydride) citrate) and poly(ethylene glycol) diacrylate copolymers for biomedical applications, and the potential application of tunable biomaterials in personalized medicine [121]; or even post-decellularized printing of cartilage extracellular matrixes [122]. With 3D bioprinting, it is also possible to develop and test new drugs or cosmetics without the need for animal or human testing. In the costly and sometimes risky traditional testing methods, up to 90% of potential medicine drugs are deemed unsuitable for final production. Therefore, it is more advantageous to test medicine drugs on dedicated tissue or using so-called organs-on-chips, where individual cells are arranged in a similar manner and proximity to that of actual organs [123,124]. Similarly, diseases, vaccines, and new cosmetics can undergo testing without relying on animal models [90,91,125]. Usually, this type of research includes mathematical modelling and theoretical design of 3D printing, emphasizing the importance of proper printing parameters to successfully print desired CAD files [126], and of tissue engineering and regenerative medicine [2], in drug discovery, and the importance of the development of implants and scaffolds, with a focus on creating complex 3D structures, ensuring cell viability, and personalizing medical treatments.

Given the substantial body of research on various aspects of 3D printing, there is significant potential for integrating ecological principles into the field. This intersection offers a wide range of opportunities to develop innovative and sustainable solutions. By advancing research on eco-friendly 3D-printed materials, energy-efficient 3D printing processes, and 3D printing-related waste reduction, researchers can contribute to more sustainable manufacturing practices. This eco-centric approach not only fosters environmental stewardship but also leads to cost-effective and efficient production methods.

### 3.3. Exploring Future Applications and Directions: Eco-Potential of 3D and 4D Printing

In the future, the development of potential applications and expanding the utilization of additive manufacturing in the context of ecology or the environment can lead to numerous benefits across various industries. Experimental design and prototyping can allow the utilization of 3D printing to create custom-designed experimental setups and prototypes for ecological studies. 3D printing can recreate scaled models of habitats and habitat reconstruction, including landscapes, forests, or aquatic environments. For example, scientists might create a 3D-printed model of a coral reef to study how changes in ocean temperature affect coral bleaching and fish populations. Ecologists can use 3D printing to create physical representations of terrain or landscapes based on geographic data to validate species distribution models (SDMs) and assess the accuracy of predictions. For instance, researchers might print topographic models of mountainous regions to study how elevation and slope affect the distribution of alpine plant species. 3D printing can enable ecologists to visualize complex ecological networks, such as food webs or interaction networks between species, to identify key species, trophic relationships, and ecosystem dynamics. As an example, scientists might print a three-dimensional model of a wetland ecosystem to study the flow of energy and nutrients between different organisms (economy of nature). Further, 3D-printed models can be used as educational tools to engage students and the public in ecological concepts. For instance, educators might use 3D-printed models of endangered species’ habitats to teach about conservation biology and the importance of preserving biodiversity. Similarly, 3D printing can be used as an educational tool that allows archaeologists and paleontologists to reconstruct fossils, artefacts, and ancient landscapes. As an illustration, scientists might print replicas of fossilized dinosaur tracks to analyze dinosaur behavior and habitat preferences.

By harnessing the power of cutting-edge technologies and aligning them with fundamental ecological principles and values, there is a lot of potential to foster a symbiotic and harmonious relationship between humanity and the natural environment. Through this integration, we can pave the way for innovative solutions that not only address pressing environmental challenges but also promote resilience and regeneration within ecosystems. In the future, additive manufacturing could be used to fabricate biomimetic materials inspired by natural ecosystems. Particularly, 4D printing (an advancement of 3D printing technology that adds the dimension of time to the process) can potentially improve ecology by creating materials that change over time in response to stimuli, are compostable, adapt to environmental changes, and have applications in sustainable agriculture and biomimetic designs. These materials would mimic the properties and functionalities of natural materials, such as self-repair, self-cleaning, and self-adaptation to environmental conditions. By harnessing the principles of biomimicry, these materials could contribute to sustainable construction, infrastructure, and product design. On the other hand, advances in bioprinting technology could enable the fabrication of living tissues, organs, and even entire ecosystems. Bioprinted materials containing living cells, microorganisms, and biomolecules could be used for ecological restoration projects, such as reforestation, wetland rehabilitation, and coral reef restoration. These bioengineered ecosystems would enhance biodiversity, ecosystem services, and resilience to environmental stressors. Future developments in 4D printing could lead to the creation of smart sensors and devices capable of monitoring and managing ecosystems in real-time. These sensors could be embedded in 3D-printed structures and materials to detect changes in environmental parameters, such as temperature, humidity, soil moisture, and air quality. By providing continuous data on ecosystem health and dynamics, these smart systems would enable more effective conservation and management strategies. 4D printing could also be applied to create adaptive infrastructure systems that respond dynamically to climate change and extreme weather events; 3D-printed seawalls and flood barriers could adjust their shape and configuration in response to rising sea levels and storm surges, providing enhanced protection to coastal communities and ecosystems. Similarly, 4D-printed green roofs and urban landscapes could regulate temperature, manage stormwater runoff, and promote biodiversity in cities. In the future, 3D and 4D printing technologies could enable the design of symbiotic structures and systems that facilitate harmonious interactions between humans and nature. For example, 3D-printed urban green spaces and vertical gardens could improve air quality, mitigate urban heat island effects, and provide habitats for wildlife in densely populated areas. These green infrastructures would promote ecological connectivity, biodiversity conservation, and human well-being in urban environments. However, responsible use of advanced 4D and 3D printing technologies needs educational experiences that deepen understandings of ecological concepts and environmental stewardship. Virtual reality (VR) and augmented reality (AR) simulations, combined with 3D-printed models and interactive displays, would allow students and the public to explore such topics. These educational tools would foster ecological literacy, empathy for nature, and informed decision-making for sustainable development, together with technological knowledge concerning additive manufacturing. Overall, the future of 3D and 4D printing dedicated to ecology holds promise for transformative innovations that enhance ecological sustainability, biodiversity conservation, and human well-being on a global scale.

## 4. Unecological Ecology of 3D Printing: Challenges and Solutions

3D printing presents a multitude of advantages, notably in the realm of sustainability and resource efficiency. By its very nature, 3D printing drastically reduces the consumption of raw materials, waste generation, and energy usage, with potential reductions of up to 90% across these parameters [33,34,127,128]. This efficiency translates to significant cost savings of approximately 15% to 60% compared to traditional manufacturing methods [3,33,34,129]. Furthermore, the environmental benefits extend to reduced greenhouse gas emissions, with an approximate 41–64% decrease when employing 3D printing technologies [33,34,100]. This reduction is attributed to the streamlined production process and the elimination of certain manufacturing steps that contribute to emissions. Additionally, the localized production capabilities of 3D printing favor regional markets and diminish the need for long-distance transportation of bulky products, further reducing carbon emissions associated with transportation. Despite its numerous benefits, 3D printing also raises several concerns and challenges that need to be addressed.

### 4.1. Trolls in 3D Printing: Materials and Wastes

While 3D printing reduces material waste compared to traditional manufacturing methods, it still produces waste in the form of non-recyclable materials through failed prints, the use of consumables, and the printing process itself, such as material powders, the use of new composite filaments, and chemicals [130,131,132,133]. Similarly, 3D printers can increase waste and pollution through the production of ultrafine particles (e.g., during selective laser sintering (SLS) and fused deposition modelling (FDM), residues from cleaning processes, untreated plastic waste, and emissions from thermal degradation. Certain 3D printing materials and processes involve the use of toxic substances, such as heavy metals or chemical additives. Improper handling or disposal of these materials can lead to soil and water contamination, posing risks to ecosystems and human health. Some 3D printing processes involve the use of chemicals, such as resins and solvents, which can emit volatile organic compounds (VOCs) and other harmful pollutants into the air. For example, 3D printing leads to the generation of alcohol/resin mixtures that can unintentionally generate small plastic particles [134] or other ultrafine particles [135,136], potentially causing environmental issues and public health risks. 3D printers themselves have a limited lifespan and may contribute to electronic waste (e-waste) when they reach the end of their useful life. The disposal of 3D printers, along with associated electronic components and peripherals, can pose challenges for responsible recycling and disposal practices. The waste generated from 3D printing operations often lacks efficient recycling avenues. Consequently, a considerable portion of this waste inevitably finds its way into landfills, contributing to environmental degradation and resource inefficiency, exacerbating the issue of plastic pollution and hindering the sustainability of 3D printing practices. The Circular Economy Action Plan and several existing EU directives are relevant to aspects of 3D printing, its waste management, and promoting their recycling and re-use. The Waste Framework Directive (2008/98/EC) establishes a legal framework for waste management and sets priorities for waste prevention and recycling [137]. It requires member states to take measures to ensure that waste is recovered or disposed of without endangering human health or harming the environment. While the Waste Electrical and Electronic Equipment (WEEE) Directive (2012/19/EU) primarily focused on electrical and electronic equipment waste, some aspects of 3D printing could fall under its purview (as shown above) [138]. Similarly, the Packaging and Packaging Waste Directive (94/62/EC) [139] or Single-Use Plastics Directive (2019/904/EU) [140] both aim to harmonize national measures on the management of packaging waste and include provisions for recycling and recovery targets. The scientific research addressing these challenges includes, apart from the obvious ones (optimization of the printing process and material used, innovative recycling and reuse technologies), the design of a new 3D printers with integrated supply systems [141,142]. This is partially dedicated to recycling plastic materials and promotes the three ecological R’s (Reduce, Reuse, and Recycle). Furthermore, multi-material printing has been a significant advancement in additive manufacturing, reducing material waste and improving production efficiency. The diversity of 3D printing processes can be minimized through the use of various patterns and gradient structures. This is particularly crucial for high-explosive materials, as it can result in significant advantages in terms of safety, cost reduction, waste reduction, and flexibility [143]. Multi-material printing using direct ink writing (DIW) for creating sustainable structures by combining a variety of materials such as ceramics, metals, polymers, and carbon, opens up possibilities for applications in energy storage, lightweight composites, and sensors [144]. Similarly, natural fibers have received attention in the area of 3D printing as more ecologically acceptable methods of manufacturing [145,146,147,148,149]. Natural fiber-reinforced thermoplastics are less harmful to the environment when compared with thermoplastic materials that release hazardous gases. Typically, wood, seeds, grass, jute, bamboo, sisal, oil palm and sugar palms, pineapple, and bananas are a few examples of natural fibers used for biopolymer composites. The absence of a well-defined end-of-life (EoL) processing system poses another significant challenge that warrants in-depth research. Many 3D printed objects are difficult to recycle due to the complex mixtures of materials used and their layer-by-layer construction. Understanding the full lifecycle of 3D-printed materials, from production to disposal, is crucial for mitigating environmental impact and maximizing resource utilization [150,151].

### 4.2. Trolls in 3D Printing: A Culture of Disposability

The widespread availability of 3D printers for entertainment purposes and the decreasing cost of entry-level models have led to an increase in the usage of plastics, rather than a reduction. This accessibility encourages experimentation and frequent printing, often without consideration for the environmental impact of the materials used. Users may default to traditional plastic filaments, rather than biodegradable PLA or recycled PETG, due to availability, cost, and printing parameters. The ease and speed of 3D printing can potentially contribute to overconsumption and ‘a culture of disposability’, where products are printed on-demand and discarded once they lose appeal or functionality, rather than being repaired or reused. The ease of 3D printing can blur the lines between necessity and desire, encouraging impulsive consumption driven by novelty rather than genuine need, and thus contributing to waste generation and environmental degradation. Indeed, the global production of plastics is increasing, from 9.2 billion tons in 2017 to a projected 34 billion tons by 2050. The next generation (within 33 years) will produce 12,000–13,000 Mt of plastic, and yearly consumption will reach 37–40 kilos of plastic per person worldwide [152]. This surge in plastic consumption exacerbates pollution in natural ecosystems, as much of the plastic is discarded and burned, but only a small fraction is recycled. In response, the European Union (EU) adopted a Strategy for Plastics in 2018 as part of the plan for a circular economy. The strategy sets goals for redesigning plastics, extending their lifespan, and increasing recycling rates to minimize waste. It mobilizes all stakeholders along the plastic value chain, from designers and producers to brands, retailers, and recyclers. One of its key targets is that ‘by 2030, all plastic packaging placed on the EU market is either reusable or can be recycled in a cost-effective manner’. However, opponents argue that transitioning to a more circular economy and prioritizing the reuse of materials will incur significant costs. To underscore the importance of these initiatives, on 2 August 2022, the EU notified a new Standardization Request on plastics recycling and recycled plastics (C(2022)5372 of 1.8.2022), in alignment with the EU Strategy [153]. This signals new challenges for the research and innovation sector, especially considering EU Regulation 1935/2004, which emphasizes the preference for recycled materials in the community for environmental reasons, provided that strict requirements are established to ensure food safety and consumer protection [154]. This regulatory framework should spark interest in scientific research. A search of the Scopus database using the combined keywords ‘ecology’ & ‘plastic’ yields a total of 2533 papers, while combining ‘ecology’ and& ‘3D printing’ results in 83 papers. However, a search using ‘ecology’ and& ‘plastic’ and& ‘3D printing’ yields only 6 papers. These papers primarily focus on topics such as the reuse of marine plastic, bioplastic development, ecological systems, and the benefits and limitations of 3D printing, including applications like mold core making and the use of polylactic acid (PLA) for fused deposition modeling (FDM) [155,156,157,158,159,160].

### 4.3. Trolls in 3D Printing: Energy Consumers

3D printers consume electricity during the printing process. While the energy consumption of individual printers may be relatively low, when scaled up for industrial or widespread use, the cumulative energy demand can become significant. The production of 3D printing materials, particularly metals and ceramics, often involves energy-intensive processes such as mining, refining, and smelting. Overall, the choice of materials, printing parameters, and process optimization play crucial roles in determining the energy consumption associated with 3D printing processes [161]. While there are no specific directives and documents from the European Union (EU) directly addressing energy consumpion in the context of 3D printing and additive manufacturing, there are several EU policies that are indirectly applicable to the field. These include the following: (1) The Eco-Design Directive (2009/125/EC), which establishes a framework for setting eco-design requirements for energy-related products [162]. The goal is to minimize the environmental impact of products across their entire lifecycle, including aspects such as energy consumption during use. (2) The Energy Efficiency Directive (2012/27/EU), which establishes energy efficiency targets and provides guidance to improve energy efficiency across various sectors, including manufacturing [163]. These directives, while not explicitly focused on 3D printing, offer guidelines and frameworks that can contribute to the optimization of energy use and environmental performance in additive manufacturing processes.

### 4.4. Trolls in 3D Printing: Social and Legal Aspects

A disadvantage of 3D printing is the potential impact of this technology on employment. The widespread adoption of 3D printing has the potential to reduce the need for manual labor in manufacturing, leading to concerns about job displacement and the implications for the workforce, particularly in industries heavily reliant on traditional manufacturing methods [164]. The transition to 3D printing may require workers to acquire new skills related to design, software programming, and machine maintenance, potentially leaving some individuals behind due to skill mismatches [165]. Therefore, disruptions in the labor market due to the adoption of 3D printing can have broader economic implications, including income inequality and regional disparities, which may exacerbate social challenges [166].

The decentralized nature of 3D printing heralds a new era of manufacturing; however, this democratization of production also raises complex legal questions regarding liability in cases of damage caused by defective products. Determining responsibility amidst the web of stakeholders involved, ranging from the manufacturer of the product, the owner of the 3D printer, and the creator of the 3D model to the end-user, poses significant challenges. Current product liability laws may struggle to adequately address these problems. As cases involving 3D printed products arise, legal precedents will play a crucial role in shaping liability standards and establishing best practices for risk mitigation. Connecting legal liability with ecology issues entails considering the environmental consequences of product defects, including pollution, resource depletion, and ecosystem disruption. Defective 3D-printed products can pose environmental hazards, such as the release of toxic chemicals from malfunctioning parts or the production of non-recyclable waste due to printing errors. Environmental protection laws and regulations should hold manufacturers accountable for ensuring that 3D printed products meet eco-friendly standards and do not pose undue harm to ecosystems or natural resources. Beyond legal frameworks, ethical considerations should also inform discussions surrounding liability, ensuring that accountability aligns with principles of fairness, safety, and consumer protection. In a similar way, the ability to 3D print items like firearms, aerospace components, and medical devices raises concerns about security and safety. However, beyond these immediate worries lie broader ecological considerations. The materials used in such projects may be toxic and resource-intensive to produce, contributing to pollution and habitat destruction. Additionally, the frequent replacement and disposal of high-tech parts can generate electronic waste, challenging waste management systems. Further, the advent of 3D scanning technologies has enabled the creation of highly detailed digital replicas of individuals, organs, and other objects, revolutionizing fields such as medicine, entertainment, and manufacturing. However, this technological advancement brings forth a myriad of ethical considerations. Without explicit consent, the creation of digital replicas infringes upon individuals’ right to control their own image and personal information. Furthermore, ownership of digital representations is often ambiguous, leading to potential exploitation and misuse of personal data. Up to now, it has been unclear who owns the rights to a digital replica: the individual whose likeness is replicated, the entity or organization that commissioned the scan, or the manufacturer of the scanning technology. These ethical dilemmas underscore the need for robust legal frameworks and regulatory guidelines to safeguard individuals’ rights in the realm of 3D scanning, including informed consent of individuals, image rights, the right to privacy, and data security. Addressing the ecological implications of creating replicas using 3D scanning technologies requires a holistic approach that considers material sourcing, waste management, and the environmental impact of printing processes. The production of replicas often requires specialized materials tailored to mimic the properties of human tissues or organs. The disposal of this waste presents environmental challenges, particularly if the materials are non-biodegradable or hazardous. Improper disposal of 3D printing waste can lead to pollution of soil, waterways, and ecosystems, posing risks to wildlife and human health. The recycling or repurposing of printing materials can mitigate waste generation, but it requires infrastructure for material recovery and processing, which may not always be available or economically feasible. Balancing the ecological footprint of these applications with their medical benefits is essential for sustainable healthcare practices.

### 4.5. Trolls in 3D Printing: The Ecological Paradoxes

In conclusion, these examples illustrate how the phrase ‘Unecological ecology of 3D printing’ encapsulates a paradoxical relationship between environmental concerns and technological advancements, prompting reflection on the unintended consequences of human innovation on the natural world. This ecological paradox highlights the inherent contradiction in promoting ecological principles, such as the use of more eco-friendly technologies like 3D printing, while engaging in practices that undermine environmental sustainability. The need to address paradoxes within the field of ecology, including the potential environmental impacts of emerging technologies like 3D printing, has resulted in the emergence of new concepts (oxymorons), not only of philosophical significance, e.g.,:

(1) Sustainable Destruction, which highlights the paradox of engaging in activities that are intended to be environmentally friendly but result in ecological harm. An example of this is 3D printing. While 3D printing boasts efficiencies in material use and the potential to reduce waste through precise manufacturing, the production of non-recyclable 3D prints (e.g., certain types of photopolymers) contradicts these benefits. Moreover, some 3D printing processes involve the use of toxic chemicals or materials that pose risks to ecosystems and human health when disposed of improperly, highlighting the unintended consequences of adopting seemingly sustainable practices without considering their full lifecycle impacts.
(2) Green Pollution, which underscores the paradoxical notion of environmental degradation occurring under the guise of eco-friendly practices. This is exemplified in the emissions of ultrafine particles (UFPs) from 3D printers, often lauded for their ability to create eco-friendly products. Released nanoparticles can harm indoor air quality and pose health risks to humans. Furthermore, the disposal of unused or expired printing materials, such as resin cartridges or filament spools, adds to electronic waste (e-waste) accumulation, further exacerbating environmental concerns.
(3) Eco-Unfriendly Technology, which highlights the paradox of technologies that are marketed as environmentally beneficial but have negative impacts on ecosystems or natural resources. Although 3D printers are celebrated for making manufacturing more accessible and reducing waste, the energy required to power these machines, especially for industrial-scale operations, can be substantial. This energy demand often relies on non-renewable sources, thus contributing to carbon emissions and exacerbating climate change, contradicting their eco-friendly characteristics. This paradox extends beyond energy consumption to include the extraction and processing of raw materials. For instance, while bioplastics like PLA are derived from renewable sources like corn starch, the agricultural practices required to cultivate crops for PLA production can have adverse environmental impacts. These may include habitat destruction, soil erosion, and the use of fertilizers and pesticides, which can harm biodiversity and ecosystems. Thus, the eco-friendliness of 3D printing materials may be overshadowed by the environmental costs associated with their production and disposal.
(4) Conservation Conundrum, which indicates the paradoxical challenges faced in balancing conservation efforts with the demands of modern society, including technologies like 3D printing. This dilemma is evident for PLA, which is biodegradable under industrial composting conditions but does not easily decompose in natural environments or standard landfills, leading to pollution. The production of bioplastics for 3D printing materials may compete with food production or contribute to monoculture farming practices, which can degrade soil health and decrease biodiversity. Additionally, the extraction of minerals and metals used in 3D printing can lead to habitat destruction and ecosystem disruption, further challenging conservation efforts.

In each of these paradoxes, the complexity of achieving genuine sustainability is underlined. They highlight the need for comprehensive approaches that consider the full lifecycle impacts of technologies, materials, and practices to ensure that efforts in ecology are reliable.

## 5. Conclusions

This exploration of ecology within the realm of 3D printing represents a critical intersection of technology, sustainability, and environmental stewardship. The title ‘LET’S PRINT AN ECOLOGY IN 3D’ encapsulates the ambition to integrate ecological principles into the fabric of 3D printing practices. The main arguments herein are centered around the need to integrate ecological, sociological, and ethical principles into 3D printing practices in order to achieve a sustainable and holistic approach to the interplay between nature and technology. 3D printing technologies can revolutionize ecology, offering innovative solutions to challenges, but research and theoretical recognition of the significant gaps in the current scientific discourse are required. A more ethical emphasis and comprehensive understanding of the complex interactions between 3D printing and ecology will result in responsible practices, such as environmental sustainability, resource efficiency, and minimization of negative impacts on society.

In the field of 3D and 4D printing, within an ecological context, several directions can move future research forward and contribute to the development of more sustainable and ethical practices. (1) Material Innovation: Research could focus on the development of new, eco-friendly materials specifically designed for 3D printing. These materials could be biodegradable, renewable, or recyclable, reducing the environmental footprint of printed products. (2) Life Cycle Analysis (LCA): Comprehensive life cycle assessments of 3D-printed products, including their production, use, and disposal, can provide insights into their overall ecological impact. This data can inform better material and process choices. (3) Waste Management and Recycling Technologies: Developing advanced methods for recycling failed prints, unused materials, and other waste generated during the 3D printing process can enhance sustainability and reduce resource consumption. (4) Energy-Efficient Processes: Research into optimizing 3D printing processes for energy efficiency, such as optimizing printing parameters and exploring alternative energy sources for printing, can minimize the carbon footprint of 3D printing. (5) Ecological Modelling and Simulation: Utilizing 3D and 4D printing to create precise models for ecological studies can advance our understanding of complex ecological systems. This could lead to better conservation strategies and management plans. (6) Ethical and Social Considerations: The development of ethical guidelines for 3D printing, such as for responsible sourcing of materials and the ethical implications of creating printed objects, can ensure that the technology is used in a socially responsible manner. (7) Regulatory Frameworks: Proposing and supporting the establishment of regulatory frameworks for 3D and 4D printing in relation to ecological and environmental standards can help guide the industry towards sustainable practices. (8) Public Education and Awareness: Educating the public about the environmental impacts of 3D printing and promoting the use of sustainable materials and practices can drive demand for eco-friendly products and influence industry standards.

In essence, the journey towards printing an ecology in 3D is not just about creating tangible objects; it is about forging a harmonious relationship between technology and nature, where ecological principles guide the design, production, and disposal of 3D printed materials. By embracing this holistic approach, we can pave the way for a more sustainable and ecologically conscious future.

## Figures and Tables

**Table 1:** The percentage of documents available in different subject areas of the Scopus database for the following keywords: E—‘ecolog*’ (total of 951,020 documents found); P—‘3D AND print*’ (total of 127,494 documents found); E + P—‘3D AND print* AND ecolog*’ (total of 461 documents found). A higher color intensity in a table cell indicates a greater significance of different subject areas in the total number of publications. Search date: 22 March 2024.

Subject Area	Agricultural and Biological Sciences	Environmental Science	Social Sciences	Earth and Planetary Sciences	Biochemistry, Genetics and Molecular Biology	Medicine	Engineering	Immunology and Microbiology	Computer Science	Arts and Humanities	Multidisciplinary	Energy	Mathematics	Chemistry	Physics and Astronomy	Business, Management and Accounting	Materials Science	Chemical Engineering	Decision Sciences	Pharmacology, Toxicology and Pharmaceutics	Economics, Econometrics and Finance	Psychology	Neuroscience	Veterinary	Nursing	Health Professions	Dentistry
**E**	25.1	21.6	7.1	6.5	5.4	5.3	4.9	3.0	2.5	2.1	2.0	1.8	1.4	1.3	1.3	1.2	1.2	1.0	0.7	0.8	1.1	1.0	0.7	0.5	0.3	0.2	0.1
**P**	1.0	1.4	1.2	0.7	4.9	5.9	25.3	0.4	8.0	0.3	0.8	1.7	2.8	6.0	9.0	1.1	19.7	5.6	0.7	1.3	0.2	0.1	0.3	0.1	0.1	0.5	0.9
**E + P**	5.3	15.6	12.2	2.3	3.1	1.5	17.9	0.8	13.4	1.1	0.8	4.2	3.1	-	4.2	3.4	5.3	3.8	0.8	-	0.4	0.4	0.4	-	-	-	-

**Table 2:** Main topics in the Scopus database as well as the number of articles (A), reviews (R), conference papers (C), and book chapters (B) for ‘3d AND print*’ AND various keywords related to modelling approaches, environment, and materials. To perform a multiple character wildcard search the “*” symbol was used. Search date: 26 March 2024.

Keywords	A	R	C	B	Examples of Application
3D ^AND^ print* ^AND^					
**modelling approaches**	13,165	1251	6417	596	Printing parameter optimization (material extrusion, layer height, infill pattern, structure, printing speed, nozzle temperature); topology (material distribution, stress analysis, enhancing strength, lightweight structures); composition (minimizing material usage, enhancing properties, reinforcement, medical drug personalization and delivery systems, tissue engineering, anatomical model generation). Process monitoring (AI-driven sensors, collection of data, optimization parameters dynamically, improving print accuracy and reliability).
mathematical ^AND^ modelling	522	30	249	23	
artificial ^AND^ intelligence	548	273	450	104	
machine ^AND^ learning	1176	219	728	66	
deep ^AND^ learning	530	56	422	14	
printing method *	1551	297	466	69	
environment *	6358	993	3299	401	Sustainable materials to reduce environmental impact (recycled, bio-based, biodegradable); circular economy (closed-loop recycling systems, minimizing waste, promoting resource efficiency, lightweight structures for reduced transportation and material usage, packaging solutions); energy-efficient processes (wind turbines, solar panels, hydroelectric generators); habitat restoration and conservation (artificial habitats for wildlife, coral reef restoration structures, erosion control and soil stabilization in degraded landscapes, sensors for air and water quality, biodiversity); waste upcycling (filament feedstock); disaster relief and humanitarian aid (shelters, infrastructure, medical supplies); urban farming and green infrastructure (hydroponic systems, vertical gardens, eco-friendly construction materials); education and awareness (interactive tools, models).
smart farming	7	9	15	4	
agriculture *	363	64	272	30	
agriculture * ^AND^ 4.0	6	6	6	3	
life cycle assessment ^OR^ LCA	156	32	10	82	
material *	36,805	4943	13,410	1722	High-performance, light structure materials for industrial applications (aerospace and automotive parts, durable consumer goods, sports and rehabilitation equipment); smart materials (textiles, medical devices, adaptive architectural elements); bioactive materials (biomedical implantable devices, tissue scaffolds, drug delivery systems); sustainable and eco-friendly materials (construction and infrastructure, green construction); functional nanomaterials (sensor fabrication); environmental monitoring (sensors, biomedical diagnostics, industrial process control); bioinspired materials (energy storage, biomimetic batteries, energy-efficient materials, sustainable energy harvesting devices); porous materials (filtration and separation, water purification filters, air filtration systems, biomedical implants); magnetic materials (actuators, sensors, and electromagnetic devices); customizable materials (orthopedic implants, consumer goods, fashion).
new material *	503	131	248	50	
plastic *	6519	465	0	186	Recycled, biodegradable filaments; packaging solutions; prototype; medical devices; consumer goods; educational tools; repair and replacement parts; sustainable construction and building parts; flexible electronics; urban furniture (parklets, benches, bike racks, and trash receptacles); prosthetics and assistive devices.
polylactic ^AND^ acid	3416	201	772	84	Sustainable consumer products (phone cases, kitchen utensils, and desk organizers); biodegradable packaging (inserts, trays, and displays for retail and shipping); educational models and toys; horticulture (planters, gardening tools, and seedling trays, disposable tableware and cutlery for events).
polyethylene	1736	93	20	25	Storage solutions (bins, organizers, shelves, durable outdoor furniture and accessories); mechanical components, automotive applications; water- and chemical-resistant lab equipment; low-cost prosthetic limbs and assistive devices for individuals.
polypropylene	531	35	156	20	Food-safe containers; durable and lightweight sport goods; industrial components (gears, bushings, conveyor belt guides); medical devices (syringe holders, specimen cups, trays).
polystyrene	414	21	91	11	Prototyping electronics (casings, covers, housings); design and planning (architectural models, landscapes, dioramas); lightweight and buoyant parts (aquatic vehicles, boats, model airplanes); designing decorative items (vases, figurines, ornaments); fabricating packaging inserts (protective packaging for delicate items).
polyurethane	1138	36	252	23	Flexible components (insoles, shoe inserts, ergonomic chairs, automotive dashboards, armrests, door panels); weather-resistant and durable outdoor elements (signage, displays, banners); orthopedic implants (limbs, braces, splints); shock-absorbing components (helmets, pads, and athletic shoes).
nylon	640	20	262	15	High-strength materials; machinery and mechanical assemblies (gears, bearings, pulleys); flexible and form-fitting medical supports; lightweight and impact-resistant drone frames, quadcopter parts, UAV components; fashion accessories.
polyvinyl alcohol	671	118	125	14	Complex geometries with water-soluble support structures; templates for casting and molding applications; dissolvable scaffolds for tissue engineering, regenerative medicine.
metal *	13,012	390	5298	431	Lightweight aerospace and automotive components (turbine blades, engine parts, brackets, engine mounts, exhaust manifolds); medical implants (hip, dental, cranial plates); tooling and molds; jewelry; military and defense (weapon mounts, armor plating, UAV parts); corrosion-resistant and heat-resistant components (oil and gas equipment, valves, fittings, connectors, marine propellers, shafts, fittings); electronics heat sinks; architecture and construction (innovative and sustainable construction projects, including facades, columns, connectors).
stainless ^AND^ steel	2498	82	703	24	Aerospace and automotive components; biomedical implants; tooling and molds; heat exchangers; marine components; electronics; architectural elements; renewable energy systems; food equipment; chemical processing components; wear-resistant coatings.
aluminum	5780	200	1462	68	Lightweight aerospace components; heat exchangers and cooling systems; electronic enclosures and components; prototyping; medical devices; renewable energy systems; high-performance; sports equipment; architectural and construction applications.
titanium	5559	372	1072	97	Biomedical implants and devices; aerospace structural components; orthopedic prosthetics; dental implants and restorations; medical instruments; high-temperature engine parts; chemical processing equipment; jewelry and luxury goods.
inconel	937	15	260	9	Aerospace engine components; turbine blades and vanes; high-temperature exhaust systems; oil and gas equipment; chemical processing components; nuclear reactor components; rocket engine components; heat treatment fixtures.
cobalt	841	57	180	8	Implants and prosthetics; dental restorations and implants; aerospace turbine components; wear-resistant tooling inserts; high-temperature engine components; magnetic materials and sensors; chemical processing equipment; jewelry and watch components.
chrome	62	2	38	2	Corrosion-resistant aerospace components; automotive engine parts; decorative and protective coatings; hydraulic and pneumatic fittings; bearing and seal components; plated tooling inserts; aerospace and defense applications; chrome-alloyed steel alloys.
copper	1893	76	818	18	Electrical conductors and connectors; heat sinks and thermal management systems; rf and microwave components; antennas; electric vehicle components; heat exchangers and cooling systems; electronics enclosures.
gold	832	111	194	23	Jewelry and luxury goods; high-end watch components; dental crowns and restorations; implants; aerospace plating and coatings; electronics connectors, decorative items.
silver	952	55	488	10	Electrical contacts and connectors; jewelry; implants and devices; aerospace plating and coatings; antimicrobial coatings; high-conductivity electronics; decorative items.
composite *	10,088	1015	2699	468	Advanced aerospace and automotive structures (aircraft components, lightweight drone wings, UAV airframes, lightweight cabin components, interior panels, vehicle chassis); biomedical implants and prosthetics (hip implants, dental crowns, knee braces, orthotic insoles); sporting goods and equipment (golf club heads, bicycle frames); energy systems (wind turbine blades, solar panel frames, battery casings, supercapacitor electrodes); marine and offshore applications (propellers, platform components); architectural elements (facades, structural supports); electronic enclosures and casings (smartphone cases, laptop enclosures); tooling and molds (injection molding inserts, die casting molds); defense and military applications (vehicle components, weapon mounts); functional prototypes and models (car prototypes, ergonomic tool prototypes); filtration, acoustic applications, thermal insulation and dissipation solutions.
carbon ^AND^ fiber	2434	220	947	101	
composite * ^AND^ carbon fiber	1206	52	501	29	
composite * ^AND^ natural fiber	195	58	82	20	
composite * ^AND^ wood fiber	76	4	25	4	
composite * ^AND^ glass fiber	367	28	169	11	
composite * ^AND^ metal * fiber	175	43	150	13	
composite * ^AND^ ceramic * fiber	113	27	69	8	
composite * ^AND^ nano *	2557	298	489	118	
composite * ^AND^ metal matrix	508	69	155	25	
biomaterial *	3255	1351	297	355	Cell, tissue, and vascular engineering; regenerative medicine (bioprinting of animal and human tissue); organ-on-a-chip devices; drug discovery and their delivery systems; development of implants and scaffolds; cartilage constructs; creating complex 3D structures; ensuring cell viability; personalizing medical treatments; biomimetic bone substitutes; implants and prosthetics; customized surgical guides; bioactive coatings; cell-laden hydrogels; neural tissue engineering
bioink *	1446	496	90	123	
hydrogel *	4398	983	411	184	
alginate	1467	92	141	35	
collagen	1440	301	85	40	
gelatin	1734	223	106	21	
fibrin	178	65	9	9	
chitosan	656	232	48	29	
cellulos *	1225	206	179	35	
synthetic ^AND^ polymer	378	252	77	59	biodegradable filaments; functional composites; high-performance structures; smart materials; conductive additives; thermoplastic printing; shape-memory constructs; medical devices; polymer blends and alloys; prototypes; polymer surface modification; polymer-based microfluidic devices.
photopolymer *	2018	235	390	62	Photopolymer resin formulations; UV-curable polymer inks; high-resolution printing; photoinitiator systems for printing; post-curing methods for photopolymer prints; biocompatible materials; toughened photopolymer resins; light-responsive composites; photopolymer-based microstructures; functional coatings; multi-material printing; nanoscale fabrication; sensors and actuators; biofabrication with photopolymer materials; photopolymer recycling and sustainability.
wax *	517	20	147	10	Support structures; high-resolution printing; multi-material printing; investment casting patterns; modelling for dental applications; lost wax casting methods; filament development; extrusion techniques; thermal properties of printed wax; new composite materials; jewelry; molds; prototyping; post-processing techniques; sustainable methods.
elastomer *	1136	93	425	19	Soft-touch filaments and material development; flexible resin formulations; printing parameters; stretchable printing techniques; biocompatible elastomer; wearable devices; elastic tissue engineering scaffolds; shape-memory materials; microstructure and surface modification; impact-resistant elastomeric materials.
graphene	1369	185	244	66	Printing inks, filaments, and techniques; multifunctional structures; nanocomposite; high-conductivity prints; flexible electronics; graphene oxide, aerogels, polymers; nanoribbon printing; sensors; biomedical applications; energy storage devices; structural components.
build *	7465	642	4051	381	Novel construction materials and components; structural optimization; energy-efficient design; large-scale 3D printing techniques for buildings; architectural features; sustainable construction; customized building designs; integration of IoT technologies; structural analysis and simulation; urban planning and infrastructure development; regulatory considerations; cost analysis and economic; viability of 3D printed buildings; temporary and emergency shelters; sustainable housing solutions.
building	3095	370	1872	211	
concrete	1472	193	730	247	
cement *	1560	157	369	152	
geopolymer *	178	43	45	24	
clay *	384	26	167	27	
ceramic *	3420	479	1070	168	Materials development; high-resolution printing; functional composite filaments; porous structures for filters and membranes; bioceramics for medical applications; nanocomposites for enhanced properties (thermal); sensors and actuators; coatings and surface modifications; electronics; multimaterial printing; energy storage devices.
porcelain	132	4	26	1	
alumina	892	20	190	15	
zirconia	578	29	76	8	
silica	1056	59	240	21	
food	1398	423	312	153	Nutrient delivery systems; food safety in 3D food printing; food designs and edible food structures; novel ingredient formulations; multimaterial printing techniques; culinary artistic creations; nutrient-rich snacks; texture-mapping and textured meat alternatives (e.g., plant-based); bioprinting of cultured meat products; personalized nutrition solutions and printing food with functional additives; decorative confections; plant-based protein products; functional food designs; development of edible printing inks; sustainability.
chocolate	64	12	25	10	
sugar *	146	12	42	9	
meat	118	65	19	10	

## Data Availability

Not applicable.
